# Clinical characteristics and outcomes of 476 mantle cell lymphoma patients aged 80 years and older

**DOI:** 10.1038/s41408-024-01204-6

**Published:** 2024-12-19

**Authors:** Simon Pahnke, Kossi D. Abalo, Sara Ekberg, Alexandra Albertsson-Lindblad, Karin E. Smedby, Mats Jerkeman, Ingrid Glimelius

**Affiliations:** 1 Department of Immunology, Genetics and Pathology, Cancer Precision Medicine, Uppsala University, Uppsala, Sweden; 2 Department of Medicine Solna, Clinical Epidemiology Division, Karolinska Institutet, Stockholm, Sweden; 3 Division of Oncology, Department of Clinical Sciences Lund, Skåne University Hospital, Lund University, Lund, Sweden; 4 Department of Hematology, Karolinska University Hospital, Stockholm, Sweden

**Keywords:** B-cell lymphoma, Epidemiology, Prognosis

Dear Editor,

Mantle cell lymphoma (MCL) is a currently incurable lymphoma subtype, with most patients relapsing after first-line therapy and a median overall survival (OS) for patients aged 70 or older of less than three years [1]. Currently, 25% of MCL patients in Sweden are over 80 at diagnosis, with no clearly established standard treatment for this age group. The number of lymphoma patients aged 80 or older is expected to double by 2040 [2]. Most studies of elderly MCL patients, however, define “elderly” as all patients over 65 or 70 years, not distinguishing outcomes for even older age groups [3–9]. Therefore, we believe that more detailed knowledge about the oldest MCL patients is needed.

Using the Swedish Lymphoma Register (SLR) and other population-based registers, we estimated progression-free survival (PFS) and OS from diagnosis, as well as concomitant disease burden, healthcare use, and causes of death, for all Swedish MCL patients aged 80 or older at diagnosis. Patients were each compared to 6–10 healthy comparators from the Swedish total population, matched for year of birth and sex.

In total, 476 MCL patients aged 80 or older were identified, diagnosed between January 1, 2000, and December 31, 2019 (Fig. S1). Most patients, 60%, were aged 80–84 (*n* = 285), with 31% aged 85–89 (*n* = 149) and 9% over 90 years of age (*n* = 42) (Table S1). The majority were male (67%) and were diagnosed with stage III (16%) or IV (58%) disease. Nearly three-quarters had a pre-existing comorbidity as measured by the Charlson comorbidity score (CCI) (73%, 349 of 476), and 37% (175 of 476) had a CCI score of 2 or higher (Table S2) [10]. Common comorbidities included prior malignancy (37%), coronary heart disease (27%), pulmonary disease (12%), and cerebrovascular disease (11%).

Four out of five patients received active oncological treatment (82%, 371 of 455 patients) (Table S3). Forty-nine patients (11%) were initially managed using a watch-and-wait approach, with 32 of these eventually receiving treatment after a median time of 6.1 months from diagnosis (range 2.8 months to 3.1 years).

Patients had shorter median OS compared to population comparators (1.5 years, 95% CI 1.2–1.9, vs. 5.1 years, 95% CI 4.9–5.3) (Fig. 1). Patients and comparators survival rates at 1, 2, and 5 years are shown in Table S4. As expected, OS for patients aged 80 or older was shorter than that of patients aged 65–79 years and 21–64 years, respectively (Fig. S2).

**Fig. 1:**
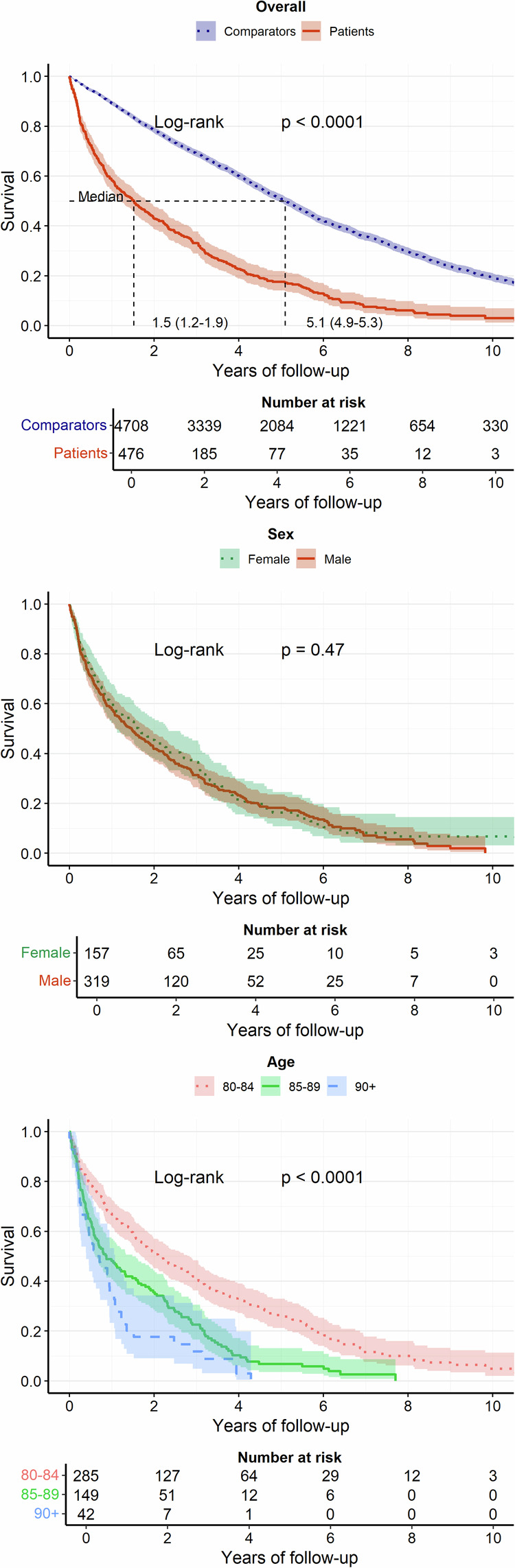
Overall survival (OS) in years, with 95% confidence intervals, for patients and matched comparators (top panel), and OS in patients stratified by sex (middle panel) and by age at diagnosis (bottom panel).

The median PFS for all patients aged 80 or older was 0.8 years (95% CI 0.7–1). No difference in OS or PFS was observed by sex; however, increasing age, CCI, performance status (ECOG), and disease stage were associated with shorter OS (Figs. 1–2) and PFS (Fig. S3). The median OS for watch-and-wait patients was 2.7 years from diagnosis (95% CI, 1.6–4.1), while median OS for patients who did not receive any treatment was 0.5 years (95% CI, 0.3–1.0).

**Fig. 2:**
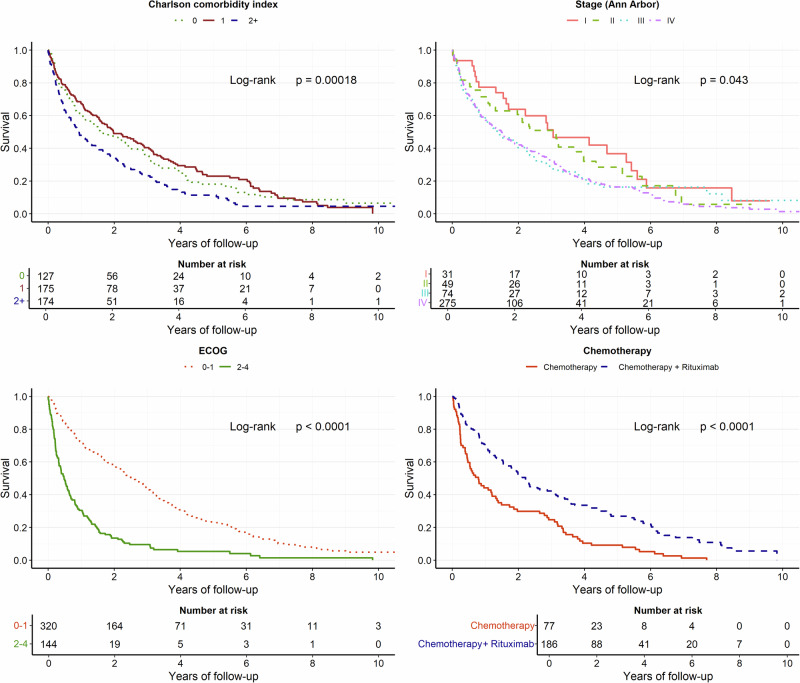
Overall survival in patients, stratified on Charlson Comorbidity Index (CCI), disease stage (Ann Arbor) at diagnosis, performance status (ECOG), and first-line chemotherapy treatment with or without rituximab.

Two-thirds of patients (68%, 307 of 455) received first-line treatment with CIT (chemoimmunotherapy = chemotherapy ± rituximab (R)), and 64% (196 of 307) of those also received rituximab (Table S3). The most common CIT regimens were bendamustine (*n* = 121, 25%), chlorambucil (*n* = 75, 16%), and CHOP (*n* = 58, 12%).

Median PFS for the different CIT regimens were: any CIT 1.1 years (95% CI, 0.9–1.1), bendamustine 1.8 years (95% CI, 1.3–2.8), chlorambucil 0.8 years (95% CI, 0.5–1.2), and CHOP 1.0 years (95% CI, 0.7–1.8) (Fig. S4). Median OS for the same regimens were: any CIT 1.5 years (95% CI, 1.3–2.0), bendamustine 2.2 years (95% CI, 1.7–3.4), chlorambucil 1.1 years (95% CI, 0.8–2.1), and CHOP 1.2 years (95% CI, 0.8–2.3), (Fig. S4).

CIT treatment including rituximab resulted in a median PFS of 1.8 years (95% CI 1.3–2.3), and median OS of 2.2 years (95% CI 1.8–3.0), compared to chemotherapy without rituximab, with PFS of 0.6 years (95% CI 0.4–0.7, Figure S3), and OS of 0.8 years (95% CI 0.5–1.4, Fig. 2). Radiotherapy was the sole primary treatment for 5.5% (*n* = 25) of patients (Table S3). Median PFS for these patients was 1.4 years (95% CI, 0.7–4.3), and median OS 4.0 years (95% CI, 2.2–n.a.).

More than 80% of patients responded to first-line treatment (Table S5). Complete response (CR) was achieved in 41%, partial response (PR) in 41%, stable disease (SD) in 6%, while 12% had primary progressive disease (PD). Half of the patients without at least a PR to first-line treatment (54%, 21 of 39) received no further treatment.

Out of 112 patients with relapsed or progressive disease, second-line treatment was administered to 86% (97 of 112) (Table S3). Most patients received CIT (67%, 65 of 97), while radiotherapy was given as the only treatment for 24% of patients (24 of 97). Best response achieved was CR in 19%, PR in 31%, SD in 14% and PD in 36%. More than half of the patients who did not achieve at least a PR (56%, 18 of 32) received no further treatment.

Less than two-thirds of patients were treated at relapse after second-line treatment (61%, 36 of 59 patients), with two-thirds (26 of 36) treated with CIT and one-third (10 of 36) with radiotherapy only.

To assess the impact of MCL diagnosis on healthcare use, we compared specialist outpatient visits, hospital inpatient stays, and inpatient days between patients and matched comparators, using data from the Swedish national patient register (Table S6) [11]. In the first year after MCL diagnosis, patients had five times more outpatient visits (IRR 5.5, 95% CI 4.8–6.3), seven times more inpatient stays (IRR 7.1, 95% CI 5.9–8.5), and five times more hospital days (IRR 5.3, 95% CI 4.1–6.9) than population comparators. In years two to five, patients had 50% more outpatient visits (IRR 1.5, 95% CI 1.1–2.0) and 30% more inpatient stays (IRR 1.3, 95% CI 1.1–1.5), while hospital days were similar (IRR 1.0, 95% CI 0.8–1.2). For the 20% of patients surviving more than five years, outpatient visits were instead nearly half those of comparators (IRR 0.6, 95% CI 0.4–0.9), and inpatient visits and hospital days were similar. The lower healthcare use at five years from diagnosis and beyond suggests that, although the MCL diagnosis and treatments are associated with a significant increase in healthcare use during the first five years after diagnosis, the general health of long-term survivors seems to be at least as good as the general population of the same age.

The cause of death for MCL patients was then analysed using data from the Swedish national cause of death register, finding that more than two-thirds of deceased patients (69%, 274 of 397) had died from lymphoma [12] (Fig. S5). This proportion was consistent across age groups but slightly higher among females, who were less likely to die from other causes.

Lymphoma being the cause of death for the majority of MCL patients aged 80 years or over emphasizes the need for continued efforts to improve therapies for this group. Bendamustine + rituximab was in a randomized trial associated with fewer severe adverse events, and longer PFS but not OS compared to CHOP + rituximab [13]. However, as the majority of patients in the study were below 70 years of age, it is uncertain if these results are equally valid for the oldest MCL patients. The imbalances in patient and disease characteristics between groups treated with different CIT regimens in our study prevent us from fully comparing outcomes by treatment regimen. In a previous study from our group on all ages, OS for R-bendamustine-treated MCL patients aged >70 years was superior in univariate analysis compared to R-CHOP-treated patients, while there was no difference when adjusting for differences in patient and disease characteristics [14].

The combination of Bruton’s tyrosine kinase inhibitor (BTKi) ibrutinib and R-bendamustine prolonged PFS for patients aged 65 years or older in the SHINE trial, but with increased risk of grade 3 or 4 adverse events, and no OS benefit for patients with high-risk MIPI [15]. In our study, 94% of patients were high-risk according to MIPI, and any clear benefit of combining ibrutinib and CIT for this group remains unproven. Ongoing randomized phase III studies ENRICH (ISRCTN 11038174) and MANGROVE (NCT04002297) are expected to provide important information on if chemotherapy-free approaches, of combinations of BTKi and rituximab, can be more beneficial options for elderly MCL patients.

A major strength of this study is the use of a large nationwide cohort, with nearly complete coverage of elderly MCL patients in Sweden. However, due to the observational nature of the data, some important limitations include the restricted possibilities to adequately compare outcomes between different treatment regimens, a lack of data on chemotherapy regimen dose modifications, the less strictly defined response evaluations in the SLR compared to clinical trials, and that the data on biologic disease characteristics is limited. MIPI was only available for a subset of pts and there is a lack of data on Ki-67 or TP53. The cohort include also patients treated in the pre-rituximab era and therefore not all patients were treated according to what would now be considered current standard of care, Novel treatments such as BTKi and lenalidomide were generally not available in Sweden for the studied population during the study period, and only three patients were treated with lenalidomide and six with BTKi (Ibrutinib) in any line of treatment. An analysis of the efficacy of these agent was therefore not possible. Given the rapidly evolving treatment landscape, this somewhat limits the clinical relevance, and the results should be read with these limitations in mind. We do however believe that, given the current lack of data regarding outcomes for this oldest group of patients, the results we present could still be of much value as more data on treatment of elderly patients with novel agents begin to emerge.

In conclusion, our study shows that the majority of MCL patients aged 80 years or older receive and respond to primary treatment. The effect of treatment is however short for most patients, and median OS is less than a third that of a matched healthy population. Encouragingly though, long-term survival is achievable, with one in five patients being alive at 5 years after diagnosis.

## Supplementary information

Supplemental material

## Data Availability

The data for this study have been extracted from Swedish nationwide registers, as detailed in the methods section. Access to these data can be obtained in accordance with European and national data protection laws. Interested researchers can contact the corresponding author (SP, simon.pahnke@medsci.uu.se) or the principal investigator (IG, ingrid.glimelius@igp.uu.se) for collaborative research projects, provided they do not overlap with ongoing projects.
