# Supplementary material for: Clinical characteristics and outcomes of 476 mantle cell lymphoma patients aged 80 years and older
Source: Blood Cancer J. 2024 Dec 19;14(1):222. doi: 10.1038/s41408-024-01204-6 (PMC11655513; doi:10.1038/s41408-024-01204-6)
Supplement: Supplementary file 1 — Supplemental material [file 41408_2024_1204_MOESM1_ESM.pdf]

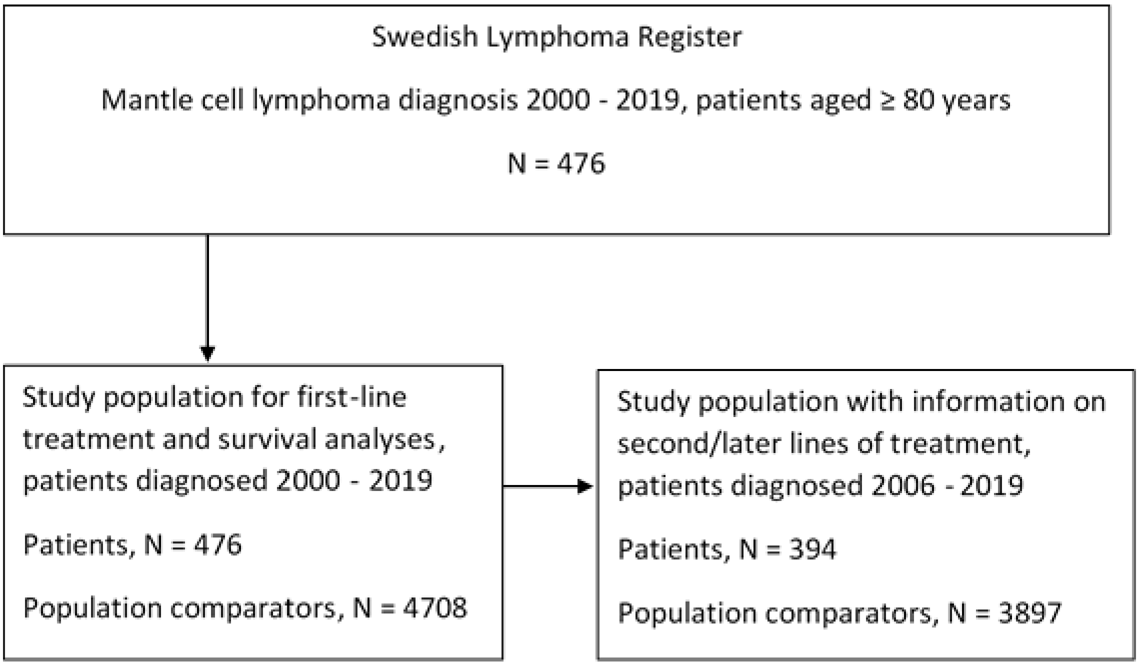


**Figure S1.**
Flowchart presenting the inclusion and exclusion of 476 patients aged 80 years or older, diagnosed with Mantle cell lymphoma in Sweden between 2000 and 2019, and 4708 population comparators, matched for year of birth and sex, at the corresponding patient's year of diagnosis.

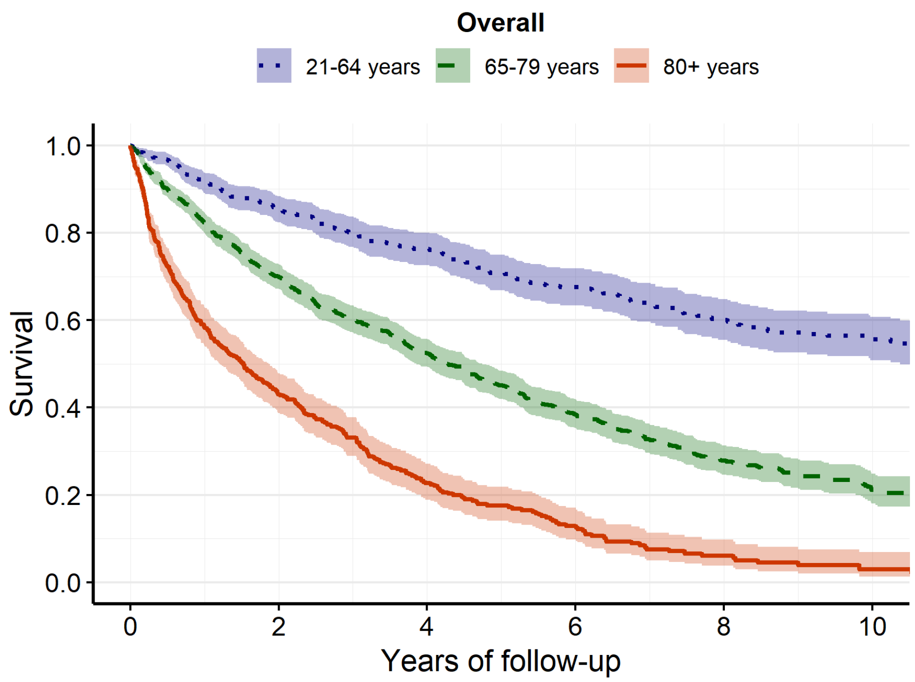


**Number at risk**

| | 0 | 2 | 4 | 6 | 8 | 10 |
|---|---|---|---|---|---|---|
| 21-64 years | 552 | 427 | 330 | 254 | 185 | 131 |
| 65-79 years | 1046 | 665 | 431 | 250 | 130 | 63 |
| 80+ years | 476 | 185 | 77 | 35 | 12 | 3 |

Years of follow-up

**Figure S2.**
Overall survival (OS) for patients, with 95% confidence intervals, stratified by age group at diagnosis.

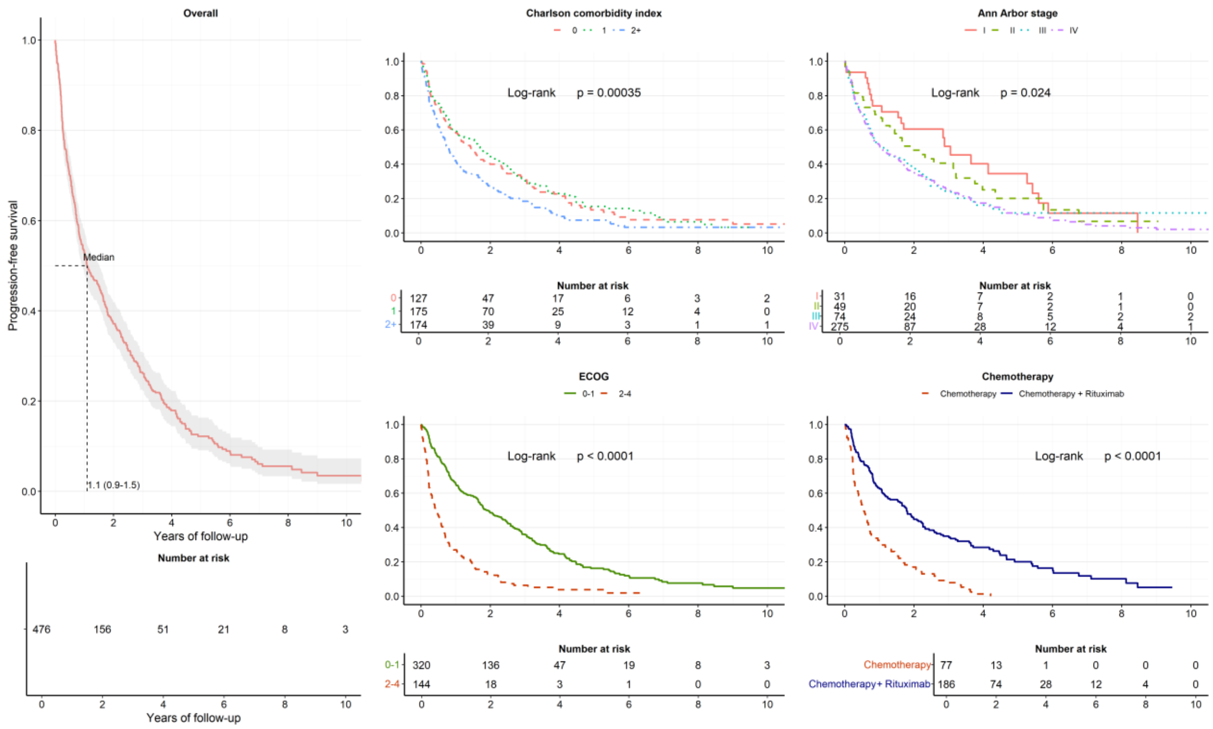


**Figure S3**.

Overall progression free survival (PFS), and stratified on Charlson Comorbidity Index (CCI), disease stage (Ann Arbor) at diagnosis, performance status (ECOG), and firstline chemotherapy treatment with or without rituximab.

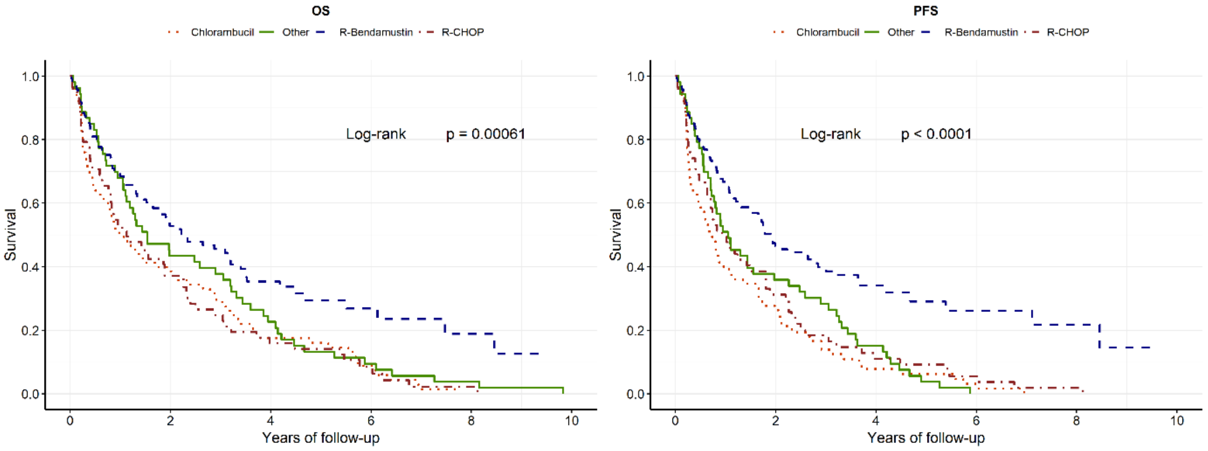


**Number at risk** (OS)

| | 0 | 2 | 4 | 6 | 8 | 10 |
|---|---|---|---|---|---|---|
| Chlorambucil | 75 | 28 | 12 | 6 | 0 | 0 |
| Other | 53 | 23 | 12 | 5 | 2 | 0 |
| R-Bendamustin | 121 | 56 | 23 | 10 | 4 | 0 |
| R-CHOP | 58 | 21 | 9 | 4 | 1 | 0 |

Years of follow-up

**Number at risk** (PFS)

| | 0 | 2 | 4 | 6 | 8 | 10 |
|---|---|---|---|---|---|---|
| Chlorambucil | 75 | 19 | 5 | 2 | 0 | 0 |
| Other | 53 | 19 | 8 | 0 | 0 | 0 |
| R-Bendamustin | 121 | 49 | 17 | 8 | 3 | 0 |
| R-CHOP | 58 | 17 | 6 | 3 | 1 | 0 |

Years of follow-up

**Figure S4**.

OS (left panel) and PFS (right panel) for patients, stratified by first line chemoimmunotherapy, in years from diagnosis.

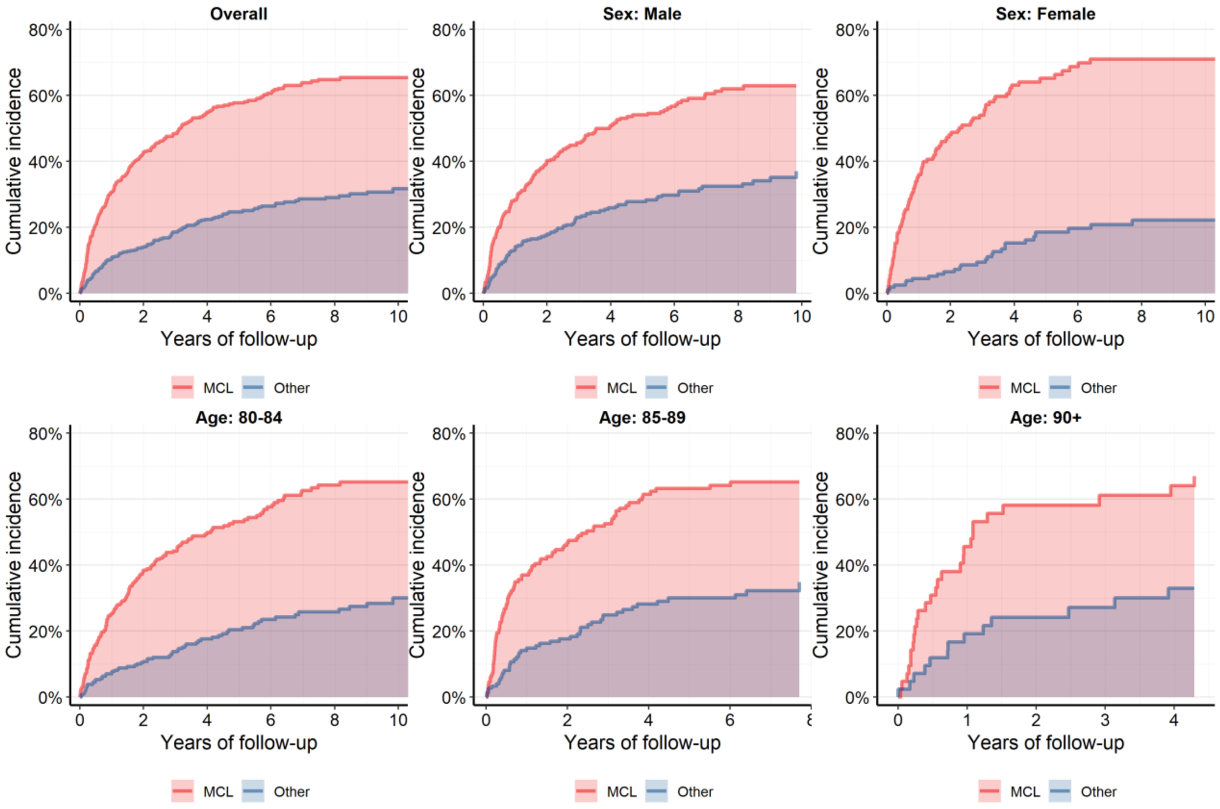


**Figure S5.**
Cumulative incidence of death due to lymphoma, accounting for competing risk of death from any other cause of death; overall and stratified by sex and age at diagnosis. The incidence curves for death due to other causes are plotted overlapping those for death due to lymphoma; i.e., in the upper left figure, the overall 10-year cumulative incidence of death is approximately 65% due to lymphoma, and 30% due to other causes.

| Table S1. Patient and cohort characteristics at primary diagnosis for all patients diagnosed 2000-2019, and for patients diagnosed 2006-2019* | | | |
|---|---|---|---|
| **Characteristics** | **Patients diagnosed 2000-2019**<br><br>**N (%)** | **Patients diagnosed 2006-2019***<br><br>**N (%)** | **Comparators**<br><br>**N (%)** |
| **All patients** | 476 | 394 | 4708 |
| **Median follow-up years (range, min-max)** | 1.3 (2.7) | 3.3 | 3.6 |
| **Sex** | | | |
| Male | 319 (67%) | 266 (68%) | 3150 (67%) |
| Female | 157 (33%) | 128 (32%) | 1558 (33%) |
| **Median age** (mean) | 83.9 (84.7) | 84.0 (84.7) | 83.9 (84.7) |
| **Age (years)** | | | |
| 80-84 | 285 (60%) | 235 (60%) | 2764 (59%) |
| 85-89 | 149 (31%) | 124 (31%) | 1540 (33%) |
| 90+ | 42 (9%) | 35 (9%) | 403 (9%) |
| **Elevated S-LD** | | | |
| Yes | 154 (32%) | 130 (35%) | |
| No | 254 (53%) | 207 (59%) | |
| Missing | 68 (14%) | 57 (6%) | |
| **Performance status** (WHO) | | | |
| 0 - 1 | 320 (67%) | 265 (67%) | |
| 2 - 4 | 144 (30%) | 117 (30%) | |
| Missing | 12 (3%) | 12 (3%) | |
| **Charlson comorbidity index** | | | |
| 0 | 127 (27%) | 90 (23%) | |
| 1 | 175 (37%) | 152 (40%) | |
| 2+ | 174 (37%) | 152 (37%) | |
| **MIPI** | | | |
| Low | - | - | |
| Intermediate | 19 (4%) | 19 (5%) | |
| High | 312 (66%) | 312 (77%) | |
| Missing | 145 (30%) | 63 (5%) | |
| **Stage** | | | |
| Ann Arbor I | 30 (6%) | 24 (6%) | |
| Ann Arbor II | 43 (9%) | 40 (10%) | |
| Ann Arbor III | 78 (16%) | 63 (16%) | |
| Ann Arbor IV | 276 (58%) | 227 (59%) | |
| Missing | 47 (10%) | 40 (9%) | |

MIPI = MCL International Prognostic Index; S-LD = S-lactate dehydrogenase; WHO = World Health Organization.

* Patients diagnosed 2006-2019 are shown separately due to data on second and later lines of treatment only being available for patients diagnosed 2006 or later.

**Table S2. Patient comorbidities at MCL diagnosis, according to Charlson comorbidity score groups (CCI) Comorbid condition All patients**

| | N =476<br>N (col %) | **CCI = 1**<br>N =174<br>N (col %) | **CCI ≥ 2**<br>N =175<br>N (col %) |
|---|---|---|---|
| Acute MI (incl. acute CHF) | 128 (27) | 26 (15) | 102 (59) |
| Peripheral vascular disease | 32 (7) | 8 (5) | 24 (14) |
| Cerebrovascular accident | 51 (11) | 14 (8) | 37 (21) |
| Dementia | 11 (2) | 1 (<1) | 10 (6) |
| Pulmonary disease | 59 (12) | 10 (6) | 49 (28) |
| Connective tissue disease | 33 (7) | 9 (5) | 24 (14) |
| Peptic ulcer | 23 (5) | 5 (3) | 18 10) |
| Liver disease | 2 (<1) | - | 2 (1) |
| Diabetes | 29 (6) | 6 (3) | 23 (13) |
| Paraplegia | 1 (<1) | - | 1 (<1) |
| Renal disease | 35 (7) | 5 (3) | 30 (17) |
| Cancer | 174 (37) | 88 (50) | 86 (49) |
| Metastatic cancer | 7 (2) | 3 (2) | 4 (2) |
| No comorbidity | 127 (27) | | |

Percentages of patients with specific comorbid conditions, total and stratified by Charlson comorbidity index (CCI). Since patients with CCI 2+ can have more than one comorbid condition, the column percentage total exceeds 100%.
Abbreviations: MI: myocardial infarction; CHF: coronary heart failure; HIV/AIDS is also included in the CCI but there were no patients with this.

| Table S3: Overview of treatment strategies | | | |
|---|---|---|---|
| | **A. First-line treatment N. (col %)** | **B. Second-line treatment* N. (col %)** | **C. Third-line treatment* N. (col %)** |
| Total | 455 | 394 | 394 |
| Any treatment | 371 (82) | 97 (25) | 36 (9) |
| Treated directly at primary diagnosis | 326 (67) | | |
| Initial watch-and-wait approach | 49 (10) | | |
| Delayed primary treatment (treated after initial watch-and-wait) | 32 (7) | | |
| Not treated | 105 (22) | | |
| Treatment information missing | 21 (4) | | |
| **Type of treatment** | | | |
| Any chemotherapy | 307 (68) | 65 (67) | 26 (72) |
| Bendamustine +/- Rituximab | 121 (25) | 24 (29) | 11 (31) |
| Chlorambucil +/- Rituximab | 75 (16) | 17 (18) | 7 (19) |
| CHOP +/- Rituximab | 58 (12) | 13 (13) | 4 (11) |
| R-CHOP/R-Cyatarabine, alternating | 9 (2) | - | - |
| Cytarabine only | 7 (1) | 3 (3) | 1 (3) |
| Other chemotherapy regimens | 37 (8) | 8 (4) | 3 (8) |
| Rituximab, all | 196 (43) | 45 (45) | 12 (33) |
| Rituximab only | 8 (2) | 6 (6) | - |
| Radiotherapy, all | 43 (9) | 27 (29) | 10 (34) |
| Radiotherapy only | 25 (5) | 24 (24) | 10 (34) |

*Information about second and later lines of treatment was only collected for patients diagnosed in 2006 or later.

| **Table S4.**<br>**Patient and comparator OS estimates at 1, 2, and 5 years.** | | | |
|---|---|---|---|
| **Time point** | **Group** | **OS (%)** | **95% Confidence Interval (%)** |
| 1 year | Patients | 58.4 | 54.1 - 63.0 |
| 1 year | Comparators | 89.3 | 88.4 - 90.2 |
| 2 years | Patients | 43.1 | 38.8 - 47.8 |
| 2 years | Comparators | 78.5 | 77.4 - 79.8 |
| 5 years | Patients | 17.6 | 14.1 - 21.9 |
| 5 years | Comparators | 51 .0 | 49.5 - 52.6 |

| **Table S5.**<br>**Response to first-line treatment** | | | | | |
|---|---|---|---|---|---|
| **Type of treatment** | **OR (CR +PR) N (%)** | **CR N (%)** | **PR N (%)** | **SD N (%)** | **PD N (%)** |
| All, n=214 | 175 (82) | 87 (41) | 88 (41) | 13 (6) | 26 (12) |
| R-Bendamustine*, n=101 | 91 (90) | 55 (54) | 36 (36) | 4 (4) | 6 (6) |
| R-Chlorambucil*, n=35 | 23 (66) | 7 (20) | 16 (46) | 3 (9) | 9 (26) |
| R-CHOP*, n=27 | 21 (78) | 5 (19) | 16 (76) | 3 (11) | 3 (11) |
| Other, n=29 | 19 (66) | 6 (21) | 13 (45) | 3 (10) | 7 (24) |
| Radiotherapy only, n=18 | 17 (94) | 12 (67) | 5 (25) | - | 1 (6) |

* this includes patients both with and without rituximab (R-) as part of the chemotherapy regimen

Abbreviations: OR = overall response, CR = complete response, PR = partial response, SD = stable disease, PD = progressive disease

| **Table S6. Incidence rate ratios (IRR) and 95% confidence intervals (95% CI) of outpatient visits (excluding routine follow-up visits for lymphoma), hospital inpatient visits, and hospital bed days during follow-up** | | | | | | | |
|---|---|---|---|---|---|---|---|
| **Type of event** | **N** | **Number of events** | **Total follow-up (years)** | **Years after diagnosis** | | | |
| | | | | **All** | **0-1 year** | **2-5 years** | **>5 years** |
| **Outpatient visits** | | | | IRR (95% CI) | IRR (95% CI) | IRR (95% CI) | IRR (95% CI) |
| Comparators | 4708 | 37024 | 20238.3 | Ref. | Ref. | Ref. | Ref. |
| MCL | 476 | 4177 | 991.8 | 2.5 (2.2 - 2.8) | 5.5 (4.8 - 6.3) | 1.5 (1.1 – 2.0) | 0.6 (0.4 - 0.9) |
| **Inpatient visits** | | | | | | | |
| Comparators | 4708 | 13874 | 20238.3 | Ref. | Ref. | Ref. | Ref. |
| MCL | 476 | 1505 | 991.8 | 2.8 (2.5 - 3.2) | 7.1 (5.9 - 8.5) | 1.3 (1.1 - 1.5) | 0.8 (0.5 - 1.2) |
| **Bed days** | | | | | | | |
| Comparators | 4708 | 105251 | 20238.3 | Ref. | Ref. | Ref. | Ref. |
| MCL | 476 | 10401 | 991.8 | 3.1 (2.5 - 3.8) | 5.3 (4.1 – 6.9) | 1.0 (0.8 - 1.2) | 0.8 (0.6 – 1.3) |
